# Exploring the role of salon professionals in identifying sex trafficking and violence victims in Indiana

**DOI:** 10.1186/s12889-024-19467-w

**Published:** 2024-07-26

**Authors:** Alexandra T. Hughes-Wegner, Andrea L. DeMaria, Laura M. Schwab-Reese, Ashley Bolen, Meagan R. DeMark, Kayra Ucpinar, Kathryn C. Seigfried-Spellar

**Affiliations:** 1 Department of Public Health, Purdue University, West Lafayette, IN USA; 2 March of Dimes, Arlington, VA USA; 3 School of Health Sciences, Purdue University, West Lafayette, IN USA; 4 Department of Speech, Language, and Hearing Sciences, Purdue University, West Lafayette, IN USA; 5 Department of Biological Sciences, Purdue University, West Lafayette, IN USA; 6 Department of Computer and Information Technology, Purdue University, West Lafayette, IN USA

**Keywords:** Salon, Sex trafficking, Intimate Partner violence, Domestic violence, Community-based interventions

## Abstract

**Background:**

Sex trafficking victims often have touchpoints with salons for waxing, styling, and other body modification services required by traffickers. Recently, some states have administered laws requiring salon professionals to receive intimate partner violence (IPV)-related training, with even fewer states mandating training on identifying sex trafficking. This study aimed to understand how salon professionals have witnessed evidence of violence, including IPV and sex trafficking, in the workplace and to explore the differences in their approach to each type of victim.

**Methods:**

In-depth interviews were conducted with salon professionals (*N* = 10) and law enforcement professionals/policymakers (*N* = 5). Content and thematic analysis techniques were used.

**Results:**

Salon professionals typically identified potential violence through signs such as bruises, odd behavior, and client disclosures, prompting them to engage in cautious conversations. Yet, few were trained to identify and intervene. Often, they responded to suspected violence by talking with the client, sharing concerns with salon leadership, directly intervening on the client’s behalf, or contacting the police. Law enforcement and salon professionals had suggestions about improving salon professionals’ recognition of and response to violence, including training on victim-focused resources, creating a safe environment, and building relationships with law enforcement. They also suggested strengthening community partnerships to increase resource advocacy and reporting.

**Conclusions:**

One-on-one salon services may provide a unique opportunity to intervene and identify victims of violence, especially when empowered through additional training and collaborative partnerships with community-oriented policing initiates. Implementing training and community-based initiatives could aid salon professionals in gaining greater confidence in knowing what to do when serving a client who is a victim of IPV or sex trafficking.

## Background

Violence against women, manifesting through intimate partner violence (IPV) and sex trafficking, remains evergreen and prevalent across the United States. According to the World Health Organization, nearly 1 in 3 women experience at least one of these forms of violence [1]. IPV perpetrators can also commit human trafficking, usually in the form of sexual exploitation [2]. Sexual exploitation is the “actual or attempted abuse of a position of vulnerability, power, or trust for sexual purposes including but not limited to profiting monetarily, socially, or politically from the sexual exploitation of another” [3]. Human trafficking in the United States (US) is nestled within a $150 billion global marketplace, where the US accounts for 52% of global human trafficking, with sex trafficking of minors accounting for the majority [4, 5]. It is estimated that 15–35% of victims trafficked for sexual exploitation are recruited by an intimate partner [2, 6–8].

Despite the prevalence of violence against women in the US, few policies support victims of these acts of violence [9, 10]. One of the more known US policies supporting women experiencing IPV is the Violence Against Women Act (VAWA) [11]. Policies often lack structure in looking at components for successful implementation, including coordination and leadership, accountability, funding, and cultural appropriateness [12]. For example, the Violence Against Women Act has loopholes that do not acknowledge protection for non-married women [11]. Even with some policies in the US, common barriers noted among IPV and human trafficking victims are evidencing fear, fear of law enforcement, shame, financial loss, and fear of danger. Victims of sex trafficking and IPV often receive little protection from the state as women, immigrants, and people of color [13]. In addition, research suggests IPV is a “push factor” (i.e., factors that make people vulnerable) for sex trafficking [14].

While many non-profit organizations are trying to raise awareness and fight against acts of violence, an uncommon ally and unique opportunity may be found in salon professionals, such as hair stylists, barbers, or cosmetologists. Sex trafficking^1^ victims often interact with salons and spas for waxing, styling, and other body modification services required by traffickers [15, 16]. Salon settings provide an opportunity to connect clients with community resources and services, as salons and related shops have been noted in the literature as community anchors and safe havens [17–21]. As seen in the DiVietro et al. [22] study, it is feasible to implement screenings in a hair salon; salon professionals could document how many clients had been abused that day, within the past year, and their lifetime. Salon professionals have long provided safe spaces and support within communities where clients return for services and interpersonal connections [23].

Health promotion and education programs are common public health practices in salons, such as screening programs for breast cancer self-examinations and the promotion of colonoscopy screenings [18, 19, 24–28]. Page et al. [29] found that clients discuss common themes with a salon professional, including family, health, identity, mental health, and women’s health. Within these themes, they found that clients are likely to talk about IPV^2^, including sexual and child abuse [29]. While this violence takes many forms, IPV and sexual violence are widespread. They can manifest in many ways, including physical or sexual violence, stalking, and psychological aggression by a current or former intimate partner.

Salon professionals can be a point of intervention for clients, meaning they can recognize clients in need, identify warning signs of mental distress, and refer clients to other resources for more formal support [30]. Salon professionals have expressed the need for additional training and support to learn how to deal with interpersonal violence conversations to reduce burnout [29]. Recently, some states, including Illinois and Tennessee, have implemented laws requiring licensed salon professionals to receive IPV-related training [31, 32]. At the time of this study, only a few states mandated training for salon professionals in identifying, intervening, or reporting victims of either sex trafficking or IPV [33, 34]. Indiana still does not require training for salon professionals in identifying victims of sex trafficking or IPV.

### Study purpose

Further research is needed to determine what training and education licensed estheticians need to feel comfortable and confident identifying sex trafficking and IPV and connecting victims to community-based services. This study aims to learn how salon professionals have witnessed sex trafficking and IPV in their salons. The secondary purpose is to determine what resources and training would aid salon professionals in identifying victims and connecting them to community-based resources. We acknowledge there is an emphasis in the literature on utilizing “survivor;” however, for the context of this paper, we will be using the term “victim” rather than “survivor” as our focus is on the point of intervention.

### Research team

The study’s research team comprised three primary investigators with expertise in human trafficking, IPV, public health, and community-based interventions. Six interdisciplinary undergraduate students supported them with training in public health, social sciences, cyber criminology, and advanced research methods. The team collected, transcribed, coded, and analyzed the qualitative data. The primary investigators monitored procedures and outcomes to confirm the reliability of the data. Researchers met biweekly to discuss coding and emergent themes. Discrepancies were settled by consensus. Additionally, throughout the research process, the team engaged in continuous reflexivity, critically reflecting on their biases and influence to ensure the credibility and integrity of the findings and direction for implications.

## Methods

Data were collected using semi-structured interviews to understand better the lived experiences of ten salon professionals in identifying and interacting with victims of sex trafficking and IPV. This study was guided by the Social-Ecological Model [35], a four-level approach to understanding violence from the individual, interpersonal, communal, and societal levels. The study was approved by the first author’s Institutional Review Board (IRB # 2021 − 1379).

### Participant characteristics

We conducted 15 in-depth interviews with licensed salon professionals (*N* = 10) and policymakers or law enforcement personnel (*N* = 5) from October 2021 to February 2022. Eligibility criteria included being 18 years or older and living and working in Indiana as a licensed salon professional, law enforcement agent, or policymaker. Those who did not meet the eligibility criteria were excluded from the study. All participants were English-speaking and were current residents of Indiana. See Tables 1 and 2 for participant characteristics. Participants were primarily recruited through direct phone calls and emails, while social media was also used to recruit and send direct messages. Before the interview, participants provided informed consent, including willingness to be audio-recorded, and completed a demographics form—all hosted on the web-based platform, Qualtrics. All participants were assigned an identification number to preserve their anonymity and separate their data from their identifiers.

**Table 1:** Salon professional participant characteristics

Item	*N* = 10
**Age (years)**	32.7 ± 10.7
**Gender Identity**	
Identified as a cisgender woman	10 (100%)
**Sexual Orientation**	
Straight/heterosexual	8 (80%)
Bisexual	1 (10%)
Preferred not to answer	1 (10%)
**Race and ethnicity**	
White, non-Hispanic	10 (100%)
**Primary Language**	
English – native speaking	10 (100%)
**License held**	
Cosmetologist	5 (50%)
Esthetician	10 (10%)
Other (educator)	1 (10%)
**Specialty**	
Lashes/Eyebrows	3 (30%)
Facials	6 (60%)
Microblading	1 (10%)
Waxing/Sugaring	5 (50%)
Education	1 (10%)
Other (Makeup, Hair, Etc.)	2 (20%)
**Years of Salon Experience**	
0–5 years	5 (50%)
6–10 years	1 (10%)
11–15 years	2 (20%)
15 + years	2 (20%)
**Setting of Salon**	
Urban	4 (40%)
Suburban	5 (50%)
Unsure	1 (10%)

Note: Data presented as M ± SD or n(%). Numbers that do not add to 100% reflect missing data or reflect participants identifying as more than one of the presented options. The age range is presented alongside the mean and standard deviation

**Table 2:** Law enforcement & policymaker participant characteristics

Item	*N* = 5
**Age (years)**	54.6 ± 12.5
**Gender Identity**	
Identified as cisgender man	2 (40%)
Identified as a cisgender woman	2 (40%)
Preferred not to answer	1 (20%)
**Sexual Orientation**	
Straight/heterosexual	4 (80%)
Preferred not to answer	1 (20%)
**Race and ethnicity**	
Identified as White, non-Hispanic	4 (80%)
Preferred not to answer	1 (20%)
**Primary Language**	
English – native speaking	4 (80%)
Preferred not to answer	1 (20%)
**Official Title**	
Policy Maker	1 (20%)
Law Enforcement	4 (80%)
**Years of Experience**	
11–15 years	2 (40%)
15 + years	3 (60%)

Note: Data presented as M ± SD or n(%). Numbers that do not add to 100% reflect missing data or reflect participants identifying as more than one of the presented options. The age range is presented alongside the mean and standard deviation

### Interviews protocol

Each salon professional interview lasted approximately 44 min (range = 28:42–52:28, M = 43:48), and each law enforcement and policymaker interview lasted about 51 min (range = 38:09–63:59, M = 50:23). All interviews were conducted virtually via Zoom or over the phone and were recorded using Otter.ai, an online transcribing tool. Upon interview completion, salon professionals, law enforcement officers, and policymakers received $60 compensation for their time in the form of an Amazon e-gift card.

To allow for flexibility and more profound insights related to the research aims, interviews reflected a semi-structured protocol better to assist the interview’s flow and time constraints. Interview questions were slightly different for salon professionals and law enforcement/policymakers. The initial questions were similar to those within these professions, allowing the participants to tell the interviewer broad information about their professional training. These opening questions created comfort and rapport with the interviewer [36]. From here, the questions diverged. Salon professionals were asked to describe their experiences with victims of sex trafficking and IPV and their thoughts on related training. These questions allowed salon professionals to describe the desired education on identification and intervention training for victims of violence. Law enforcement and policymakers were asked similar questions but focused on the current policies and if they had heard about salon professionals having experiences with victims of violence. Questions were also asked to understand better how law enforcement and policymakers thought salon professionals would respond to a policy encouraging violence-related training. The interview guides were modified within IRB guidelines to help improve content development as data were collected. For example, questions were reorganized to prioritize receiving responses to questions near the end of the guide, and questions were edited to improve clarity on what was being asked. See Table 3 for sample questions.

**Table 3:** Representative interview questions

Topic	Interview Question
Salon Professionals: Experiences with victims of sex trafficking and IPV	Do you think you have ever interacted with someone in your professional space who was a victim of sex trafficking? Do you think you have ever interacted with someone in your professional space who was a victim of domestic violence? If no, has a colleague or peer ever discussed experiencing this with you?
Salon Professional: Thoughts on training	What types of training do you think would be helpful to you related to sex trafficking and domestic violence for your field? In what ways can service providers, like yourself, help support victims of sex trafficking and violence?
Law Enforcement/Policymaker: Salons as intervention points	Have you heard about licensed salon professionals in Indiana suspecting they are interacting with victims of sex trafficking or domestic violence? ).

Note: Please contact the corresponding author to access the complete interview guides.

### Data analysis

An immersive, full content review was completed to ensure familiarity with all data, noting immediate patterns and ideas for potential codes and themes [37, 38]. Following the content review, thematic and qualitative content analyses were employed during the initial creation of the codebook to promote a diverse presentation of the data and gain insights into participant experiences and attitudes. Code development followed the adaptation of grounded theory described by Schreier [39]. The team read the transcripts and took notes on repeated topics and ideas across transcripts and unique areas. Then, the team re-read the transcripts, focusing on identifying information not captured in the notes from the first review. As we refined the codebook, the team met to discuss emerging materials and define and develop codes. After completing the framework and definitions, team members pilot-coded several transcripts and then met to discuss differences in coding. Differences were resolved through consensus, and the codebook and definitions were refined. We continued this process until coding was consistent across the team, and then the final codebook was applied to all available transcripts. As additional interviews were completed, the transcripts were reviewed and coded. While processing the additional transcripts, the team assessed whether saturation was reached when adding more material did not produce additional insight [40]. The team reached saturation near the end of data collection and then completed the interviews previously scheduled. To verify the accuracy of the codes, two independent rounds of coding were used to ensure correctness, with the second used to find missing codes or amend improperly coded sections.

Data were combined into themes and subthemes when the coding process was finalized. Theme development was data-driven and closely reflected participant responses. The research team worked together to analyze all themes and subthemes to include varying levels of value. All issues were resolved through unanimous consensus to minimize biases. The codebook and coding process were completed from February through May 2022. HyperRESEARCH 4.5.1 was used for data management.

## Results

Results are presented within the scope of the Social-Ecological Model, focusing on individual and relational experiences and the role of schools, employers, and states as communal and societal layers, and concludes with recommendations for trainings, networks, and resources. Themes and subthemes are presented below with representative quotes followed by participant gender identity (i.e., woman (w) man (m)) and profession (e.g., esthetician).

### Individual & relational experiences

#### Sex trafficking: suspicions & a lack of experience

When asked about their experience with sex trafficking, many salon professionals had no experience saying, “I do not believe I have come across that” (w, esthetician) or simply “no” (w, esthetician). Of these participants, a few assumed it might be happening but were unsure, with one participant noting, “a couple of times I suspected that it could be happening” (w, esthetician). Even though most did not have experience with sex trafficking, more than half knew how they would approach the situation, which included starting a conversation with the victim. One participant described how if she believed she identified a victim, she would take them somewhere private and “start a conversation” (w, esthetician). Although the majority agreed on starting a conversation, some were concerned about their ability to engage. One participant described how they would want to talk to the victim but said, “I don’t know what I would do because I rarely confront even the salon owner or the salon manager sometimes throughout a given day” (w, esthetician). Overall, salon professionals had little to no experience regarding sex trafficking.

#### IPV: a common experience

Most salon professionals expressed experiences with IPV, directly or indirectly, through coworkers. Some simply said “yes” in response to having direct experience (w, esthetician; w, esthetician), while another said, “I have one client that talked about it openly, and they have children together” (w, esthetician). Another salon professional stated, “There’s one that I’m dealing with right now” (w, esthetician). When describing their clientele’s experiences with violence, a majority reported both physical and verbal violence. One salon professional described an experience where a client could not cut her hair due to her abusive partner:

So one scenario being that she was asking me so bad to cut all of her hair off, but because her partner was there and was very dominant in this situation, I could not do that for her that I would normally do in this setting. But that was her main abuse that he would pull her hair and grab it of why I couldn’t do for her what I could or wanted to (w, cosmetologist/educator).

One participant said she had no experience with IPV but suspected some clients may be victims because they “had bruises, like all over them, on a regular basis” (w, esthetician). The clients themselves disclosed other forms of violence. One client “expressed that their partner has been controlling” (w, esthetician) or “he follows [the client’s] every move” (w, esthetician). Even when a salon professional did not have IPV-related interactions with clients, most would share their coworkers’ experiences. One salon professional noted their coworker having “a couple of clients” throughout their salon where they suspected violence to be at play in their personal lives (w, esthetician).

#### Identifying odd behavior and direct conversations

Client behaviors typically caused speculation among salon professionals, with one noting how she identified possible victims because “they are not acting as they typically would” (w, policymaker). Another participant elaborated on this by saying, “[I] think any hairdresser could pick up [on] any domestic violence, you can tell by the bruises or by the attitudes, or the excuses” (w, law enforcement). In response to identifying bruises, one participant mentioned:

And then they’re, they’re gonna say “no, my boyfriend’s just being an asshole,” or “my fiancé… we were just in an argument” or “I don’t know whatever,” you know, I mean, it’s gonna be a hard stretch to get them to disclose any type of abuse that’s occurring, I think (m, law enforcement).

When salon professionals were asked how they responded to finding out their clients were experiencing IPV, most said they would simply talk to them. When asked about how they would start a conversation with a client, they discussed being cautious of not crossing a line with their client and “silent talking” methods:

So we have to be careful to not cross the line. And that’s where we can do the best that we can to silently talk to them…to see if they need help or what we can step in to do because, of course, whomever we work by, there’s guidelines that we have to follow, and at the same time that we don’t want to be put into somebody else’s situation, um on the job (w, cosmetologist/educator).

This participant followed up later with, “You just hope that you ask a question that allows that person to drop their guard because, of course, that’s very hard for anybody to do to drop the wall down to a complete stranger” (w, cosmetologist/educator). Similarly, another participant would ask, “’Are you being hurt?’ You know, you can kind of lead into that, ‘Hey, are you okay, super stressed? Are there things going on at home? Feel free, this is a safe place to talk’, you know?” to try and make her clients as comfortable as possible. Many participants described how their coworkers would also similarly talk to their clients by making them comfortable and checking in on them (w, esthetician; w, esthetician; w, esthetician). While salon professionals mentioned interactions with victims of IPV in their salons, many noted being unsure of their approaches. Overall, when compared to experiences with sex trafficking, salon professionals and law enforcement had more experience with and knowledge of IPV.

### Communal and societal: the role of schools, employers, and States

When salon professionals were asked about who is responsible for enforcing the required training in these content areas, a majority expressed a preference during the time of licensing, with one professional noting:

[she] hopes every state passes to have [training] as a part of our licensing. I think everybody should go through some kind of course to know that… to be able to hear stories or talk and have a more comfortable-ness on how to approach it (w, cosmetologist/educator).

Some salon professionals stated how IPV and sex trafficking should be “part of beauty school curriculum” (w, esthetician) and “talked about in beauty school and like how to recognize when that’s happening” (w, esthetician), while another expressed wanting to “[learn], physical body movements, [and] being … [trained to] lookout for any kinds of bruising… physical ailments and then… verbal cues” ahead of entering the workforce (w, esthetician). One participant connected this requirement to others in the field, noting, “I think it would be great if it was a requirement, honestly, just like sanitation and every other thing” (w, esthetician). When the same question was asked to a police officer, he agreed the training would best be delivered “during the time of licensure … [as] salon professionals already have a lot of training…” (w, law enforcement).

Not every state has the same requirements for licensing or licensing renewal. One participant noted a time when training was once required (16 h every 4 years) but no longer is, “Unfortunately, several years ago, many years ago, [states] took away continuing education requirements from our industry. And I think that that was terrible… it should be something that is required” (w, esthetician). Another participant suggested annual training requirements for all states, “I would give a recommendation of having it every year because if you’re waiting four years, maybe you might forget it… unless companies in the state worked together to make sure people kept up on this training” (w, cosmetologist/educator). One salon professional expressed interest in post-graduate training certification programs, saying, “It would be nice to… be certified because that’s something we can go in and talk to the clients about” (w, esthetician). The participant continued to mention how certification could allow salon professionals to showcase being a safe zone for victims. Until states and industry align on requirements, professionals will continue to self-educate “I just keep trying to teach myself more things about this kind of stuff, because it does bother me” (w, cosmetologist) so that they “want to be more educated” (w, esthetician) to identify or intervene, if needed.

A recommendation could be for employers to step in, with one salon professional suggesting “each salon, each individual who may own their salon suite or something…have something [a plan] in place” (w, esthetician) for what to do if they suspected a client in danger. One participant noted the need to be protected when intervening, saying, “I guess just make sure that there’s laws in place…to support people who are willing to help so you’re not in any sticky situations being sued by a person, you know?” (w, esthetician). Law enforcement participants were much more experienced and trained in sex trafficking than salon professionals. Despite these differences, salon professionals and law enforcement agreed that training programs should address these issues during schooling. States, including Indiana, should require sex trafficking and IPV training as part of licensing.

### Training, network, and resource recommendations

#### Training

When salon professionals were asked how sex trafficking and IPV-related training should be implemented, most participants preferred in-person programs. One professional believed having training in-person makes it “more personable” (w, esthetician), while another thought it would “actually make people pay attention… [because] people are gonna remember it more than just hearing somebody talking, talking, talking [online] (w, esthetician). Another professional highlighted the associated benefit of having speakers by stating, “It’s really great when you have people come in and speak directly on the topic” (w, esthetician). Additionally, one participant, previously having worked with organizing advocacy forums, gave examples of speakers ranging “from the police department, the battered women’s shelters, [or] self-defense individuals to come in to talk to students” (w, policymaker).

Many salon professionals believed sex trafficking and IPV information was not clear, accessible, or available during their licensing training. In general, most salon professionals were interested in learning more. One participant stated, “I guess I would want to know if there any particular signs to look for because it’s so hard for me personally to judge somebody or judge their situation without them being upfront” (w, esthetician). Most echoed this idea by stating, “I would just say like exposure to understanding like, what are red flags? What do they mean?” (w, esthetician) and “I would like to see one, I guess, good ways, for sure ways, to identify it [sex trafficking or IPV] or things to look for” (w, esthetician). Related to this, one law enforcement participant mentioned, “I think any hairdresser could pick up any domestic violence, you can tell by the bruise or by the attitude, or the excuses” (w, law enforcement). Another had ideas on training, declaring, “I think if we could give them training to look for. I mean, they’re looking at some parts of the body that most people aren’t going to see, if they can look for bruising” (m, law enforcement). Most salon professionals were unsure of identifying signs but believed they could be taught signs through training.

While knowing the signs would be a necessary first step, some salon professionals explained how they would not know the next steps even if they knew them. One participant stated how they would “want to know immediately, like…who do they [the salon professionals] talk to? Who do I talk to if something is going on?” (w, esthetician) and would want to learn “how to intervene, how to talk to your client…what authorities you contact, how do you talk to somebody?” (w, esthetician). Wanting to learn how to approach the situation was a common query. One salon professional stated training “would be able to tell us what we can do, what we can’t do, you know, with[out] overstepping. What can push people away?” (w, cosmetologist) and another said, “I would want to know what is the best way to get them [the victims] help” (w, esthetician). Salon professionals also voiced concerns about knowing who to contact for help. One salon professional said, “I think that we should know as business owners what that number is to call. Is that 911? Is it, you know, a specific helpline? Like, who do you go to, to get somebody help?” (w, esthetician). Additionally, when discussing what IPV and sex trafficking-related information should be provided to salon professionals as part of training, a Title IX coordinator stated, “Give them a list of agencies to call…to help the client if the client wants” (w, policymaker). Most salon professionals did not know what to do once a victim was identified or who to contact for help.

#### Network

Professionals mentioned continuing education could benefit from partnering with community resources focused on IPV and sex trafficking. One professional expressed an idea of creating “a night of education that works with shelters or studies or know people who are working with those women, that helps teach what to look out for or what we can or can’t say” (w, esthetician). Similarly, another participant felt “we need an organization… where we can, you know, host events, host these things, you know, host awareness, and talk about it” (w, esthetician). Another participant discussed a program she began in her beauty school that included informational pamphlets, which supplied “students and salon professionals that list…things to look for, or behavior patterns that might have changed amongst their clientele” (w, policymaker). These community-based resources would allow salon professionals to continue their education to be better prepared to help IPV and sex trafficking victims.

While most salon professionals agreed on when the training should occur, they differed on who should teach these materials. Some believed local law enforcement should be involved with or without other speakers (e.g., “classes should be presented by law enforcement and a psychiatrist” (w, cosmetologist); “speakers from the police department” (w, policymaker)). Others stated that law enforcement should not be involved, believing that “having somebody that isn’t a police officer is more like I said of that psychology-based or therapy-based person” (w, esthetician). One participant agreed, saying, “It would be better if there was like a counselor who could intervene rather than just a police officer” (w, esthetician). Another participant even mentioned a need for “self-defense individuals to come in to talk to the students” (w, policymaker). Non-professional speakers, such as victims themselves, were another suggested example for speakers:

It’s always better for others to connect when you hear it from somebody that’s gone through it … to be able to hear stories, makes it true, makes it human, makes it real and raw to people and others to be able to start speaking upon it (w, cosmetologist).

Salon professionals and law enforcement agreed that required training for salon professionals would be beneficial. Many also believed this informative process should include individuals with practical experience engaging with IPV victims.

#### Resources

Along with not knowing what to do, salon professionals reported not knowing what resources are available in their communities. One salon professional declared, “More recognition on this [is needed] to be able to know the resources that you have. Because people don’t realize what resources are even out there… it would make a huge difference” (w, cosmetologist/educator). Another participant agreed, stating, “You know, we can offer support, we can offer a lending ear. But beyond that, we need to be able to know where to direct them [the victims] and how to get them more help” (w, esthetician). While many salon professionals did not know what to do or where to go for more information or help, they did offer ideas on what is needed to resolve this lack of awareness.

Most salon professionals believed they could more effectively respond to violence if they had access to community resources. One participant noted it would be helpful for victims to have access to a number that would go “[to a] department within the city, [for] … anonymous tips or whatever come in [and] having some possible way of encountering these individuals without shooting up red flags for their abusers” (w, esthetician). Additionally, another professional suggested how these anonymous tip lines could be implemented in salons by “putting [up] sign[s] in the bathroom…with a number or something” (w, esthetician) available to call for those needing specific help. Another participant supported this idea by referencing alert symbols on social media applications for victims feeling trapped in bad situations as well as “certain bathrooms, [having this] text-this-number kind of thing” (w, esthetician). On the other hand, one professional felt a hotline focused solely on reporting cases might not be as beneficial as one focused on supporting the victims. This was explained by a professional who suggested using a hotline “for people to talk, maybe therapy, [or if they] need legal advice” (w, esthetician). About half of law enforcement participants knew of different resources a salon professional or the victim could utilize. Of the resources discussed, most recommended coming to the police advising, “they need to call for a police officer” (w, law enforcement).

Additional resources professionals supported were informational pamphlets that could be handed to clients. These pamphlets could direct clients to resources within their community and outline “places that women [or men] can go if they’re abused or just something that has some general information to give someone hope that feels so lost in that situation” (w, esthetician). They also suggested how the resource pamphlet could be slipped to suspected victims in the salon as they check out. These pamphlets could direct victims towards aid while serving as continued education by informing professionals what they should look out for so they may assist those victims.

## Discussion

As the interviews revealed, experience with sex trafficking may not be common. The lack of knowledge may lead to a lack of awareness of the signs a victim could show. Many signs are not obvious in public settings; in many cases, even the victim may not see what is happening [41]. Even if a salon professional assumes they have identified a victim, the lack of experience leaves them feeling hopeless about what to do if the situation presents itself. While most had ideas about communicating with the victim as their first step, they were not confident enough in their communication skills or if sharing would be the best way to help a victim. Few mentioned getting the police involved; however, when talking to law enforcement, most advised coming to the police. Opening the communication between the police and salon professionals may foster a clearer understanding of what to do when interacting with a possible sex trafficking victim. In a similar study of sex trafficking experiences among hotel employees, most noted never having an experience or encounter; however, after training, they felt confident about what to do if ever in a situation [42]. Thus, training salon professionals in identifying sex trafficking victims and where to turn for support may result in more confidence and a greater likelihood of reporting.

Similar to other studies focusing on the role salon professionals can play in health interventions [18, 19, 22, 27, 43], clients were open in telling their violence-related stories, or stories of those close to them, with their salon professionals. Many participants noted their experiences with their clients related to IPV, including physical and verbal abuse disclosures. DiVietro et al. [22] reiterated this by having salon professionals screen their clients and found many shared being a part of abuse. Some reporting on physical abuse could be explained by intimate contact with salon professionals, such as closeness to the head and neck during a haircut or seeing bare body parts during a hair removal process. Similarly, participants mentioned it was easy for clients to open up and disclose sensitive information as they developed a friendly, comfortable rapport. Evidence from past studies noted most salon professionals feel close to their clients [19, 43]. Conversation with suspected victims can occur due to this feeling of intimacy, and communicating concern is the main form of interaction salon professionals feel comfortable with. Despite having little to no knowledge of how to interact or intervene, most salon professionals tried to give advice or direct them to the police for help.

Salon professionals repeatedly stated that even if they did recognize a possible IPV or sex trafficking victim, they would not know where to go or from whom to get help, likely because most participants noted having little to no training. While some states [31, 32, 44] require IPV training for salon professional licensure (Indiana is not one of them), no state currently requires sex trafficking education. Since Indiana does not have education and training requirements on these topics, many salon professionals we interviewed disclosed knowing little and being unaware of how to intervene. However, most salon professionals expressed building trust and rapport with their clientele, which resulted in sharing personal information [45]. The salon environment provides a chance to connect with clients who need the necessary resources [19]. Page et al. [29] found salon professionals requested more training and resources related to IPV to facilitate these conversations and reduce the emotional burnout they can experience. According to Sattler and Deane [30], when salon professionals did not know how to have tough conversations, anxiety and unpreparedness overwhelmed the workers. Furthermore, researchers have found that hair stylists who have received training in discussing family violence have increased self-confidence in their ability to refer their clients [24]. Law enforcement participants affirmed these conversational interactions and close bodily contact would allow salon professionals to identify possible victims quickly.

### Training recommendations: modality, frequency, & collaboration

The study participants provided various recommendations on the potential structure of a tailored training program, encompassing aspects such as modality and frequency, content, and collaboration. Most salon professionals preferred in-person training programs during licensing, which could create opportunities to connect with peers and professionals on these topics and victims. Programs could include expert guest speakers from various backgrounds (e.g., law enforcement, psychologists, social workers, and community leaders) for a comprehensive and inclusive approach. Some salon professionals were hesitant about having law enforcement as guest speakers due to the possibility of their presence triggering possible victims going through training. A suggested alternative could be crisis counselors and self-defense trainers. Participants believed this unique collaboration among these diverse professionals would better equip trainees with the techniques required to identify the physical and non-physical indicators of violence. Collaboration with others, such as law enforcement and policymakers, could provide pamphlets for salon professionals to reference post-training, containing information about the signs of violence (e.g., unexplained bruising or injuries, changes in behavior, hypervigilance) and resources to contact or direct their clients to.

### A change in continuing education policy

Continuing education is not mandatory in Indiana for salon professionals to renew their licenses; however, they allow salon professionals to remain up-to-date on industry trends, standards in care, and relevant industry information [34, 46]. Illinois requires at least one hour of continuing training in IPV and sexual assault awareness for license renewal [31]. A change in continuing education requirements for licensure renewal would ensure salon professionals are aware of the latest content related to IPV and sex trafficking, a common practice for many professional licenses. Our participants believed there should be requirements within their profession for re-training, such as an IPV refresher course, which would then allow salons to advertise themselves as a safe zone for clients. Post-graduation training programs could also partner with local organizations to host education nights to increase awareness of IPV and sex trafficking in their communities. Through continued education and partnership with community-based initiatives, salon professionals can be a crucial resource for victims through early abuse detection and further intervention.

### Training outcomes and products

Salon professionals had many ideas regarding the implementation of future training programs. A popular community resource idea amongst professionals was the creation of an anonymous hotline for support, similar to those offered through the National Human Trafficking Hotline and Indiana Trafficking Victims Assistance Program [47, 48]. These hotlines could be promoted in private spaces, such as bathrooms, on signs or posters for victims to report situations, or anonymously for legal-based advice. Another suggestion required salons to keep an accessible supply of informational pamphlets tailored to victims. Pamphlets would be developed in congruence with local law enforcement and community-based programs, outlining safe spaces for victims to go and directing them toward other resources within the community for further support. A program such as this, which provides training for salon professionals in identifying and intervening in IPV, currently has some success [49]. Implementing and utilizing support systems within local communities can give salon professionals the tools to help and advocate for their clients.

### Implications

The implications of these findings for policy and practice regarding training, resource allocation, and support systems for salon professionals are significant. Addressing the needs of both sex trafficking and IPV victims holds the potential for a broader impact on efforts to combat violence and support survivors. Initiatives incorporating comprehensive training programs during licensing and ongoing education can empower salon professionals to recognize and effectively address these forms of violence. This empowerment can facilitate collaboration between salon professionals, law enforcement, and local resources, enhancing the network of support available to victims and contributing to prevention and intervention efforts.

A unique opportunity arises in utilizing salon professionals as lay health workers and advocates for health and well-being. Their discreet environment, often hosting intimate salon services such as hair removal and grooming, offers a distinct opportunity to detect instances of IPV and sex trafficking, facilitating timely referrals to appropriate resources. However, many professionals expressed a lack of confidence in their ability to take action and assist victims even if they could identify them, a sentiment shared among many US citizens [50]. There is a clear demand among salon professionals for training programs that equip them with the necessary knowledge and skills to handle such situations effectively. These training programs should address individual, interpersonal, and communal aspects, guided by the Social-Ecological Model [35] and prevention strategies principles [51].

Salon professionals suggested that such training be implemented during licensing and supplemented with continued education and certification requirements [52]. Furthermore, practical support systems within salon spaces, such as anonymous hotlines and informational pamphlets, can serve as valuable resources for victims and professionals. Additionally, considering the diverse needs and challenges faced by victims of sex trafficking and IPV, it is crucial to develop evidence-based interventions tailored to their specific circumstances. By establishing collaborative networks between salon professionals, law enforcement, and local resources, communities can create a supportive environment that aids victims and enhances efforts to prevent and intervene in instances of violence.

Current resources for IPV, such as tip lines, safe zones, or notifying law enforcement, may not be available to sex trafficking victims. For instance, some victims may not have a private space without surveillance to call a tip line, so additional strategies could be employed, such as keywords or phrases a client could say to their salon professional to cue them to intervene. This strategy (e.g., angel haircut with layers) went viral on TikTok in 2022 and should be further explored [53]. In addition, sex trafficking victims are often fearful and distrusting of law enforcement, making this intervention strategy complex [13]. Some training and resources are compiled on the US Department of State’s website [54]; however, there is no evidence of the effectiveness of these programs or materials. More work is needed to design evidence-based interventions that are implemented and evaluated.

### Strengths, limitations, & future research

Little is known about salon professionals’ role in identifying and intervening in sex trafficking. We can learn from the existing literature how salon professionals have been used to promote other realms of health and how different industries identify and interact with victims of violence. Qualitative methods allowed us to explore law enforcement and salon professional perspectives. As an exploratory study, our sample size was limited. Participants’ ages varied, which allowed many different accounts from all ages and levels of experience, especially within the salon industry. While the race and ethnicity of the professionals interviewed were noted in our demographics, the demographics of the population they serve are unknown. The findings will not be generalizable geographically, demographically, or socio-economically to all women. Despite these limitations, our study contributes to the limited research surrounding salon professionals’ role in identifying violence victims.

Future research is needed to understand the perspectives of a more diverse sample of salon professionals. If salon professionals, law enforcement, and policymakers see the value of this approach, components of successful training programs need to be identified, including the topics, format, and when training occurs relative to licensure. Empowering salon professionals to identify appropriate resources and make referrals may be critical to increasing the impact of the training on practice. Additionally, future research needs to examine the lived socioemotional experiences of salon professionals. Often seen as informal caretakers, salon professionals take on an extraneous mental toll through constant intimate conversations with clientele [21, 55, 56].

An overarching theme identified in the current study was the varying perspectives on how, when, and who should implement future training with salon professionals on sex trafficking and IPV. Thus, an important research avenue is testing future training with different facilitators (e.g., law enforcement, salon professionals, advocacy groups, academics) and delivery modes (in-person, virtual, hybrid) to determine the most effective approach to training salon professionals. Our findings suggest these trainings are desired and may have a secondary positive impact on the health and well-being of salon professionals.

## Conclusions

Our study findings highlight the role salon professionals can play in identifying and addressing issues related to IPV and sex trafficking. Despite lacking formal training, many salon professionals were willing to engage with and support their clients, emphasizing the need for comprehensive training programs. The recommendations for in-person training, ongoing education, and collaborative efforts with law enforcement and community resources showcase the importance of equipping salon professionals with the knowledge and skills necessary to recognize and respond to signs of abuse and violence. By incorporating these training programs during licensing and through continued education, salon professionals can become more confident and effective in their roles as lay health workers and advocates. Furthermore, developing supportive resources, such as anonymous hotlines and informational pamphlets, can enhance their ability to assist victims. Implementing these strategies can foster a supportive environment within salons, contributing to broader prevention and intervention efforts in combating IPV and sex trafficking.

## Data Availability

Transcripts and interview guides are available by request. Contact Alexandra Hughes with your request (hughe160@purdue.edu).

## Footnotes

1 For this paper, sex trafficking was defined as human trafficking for the purpose of sexual exploitation and does not require kidnapping and/or crossing borders. Sex trafficking is referred to as the “the act (e.g., recruitment and transfer), means (e.g., fraud and abuse of power), and the purpose (e.g., sexual exploitation).” Victims of sex trafficking can be all ages and genders.

2 For this paper, IPV refers to “acts of physical violence (e.g., slapping, hitting, kicking, and beating), psychological abuse (e.g., insults, belittling, constant humiliation, intimidation), sexual violence (e.g., forced sexual intercourse and other forms of sexual coercion), and/or controlling behaviors (e.g., isolating a person from family and friends, monitoring their movements) occurring in an intimate relationship”.
